# A spatially explicit model for estimating risks of pesticide exposure to bird populations

**DOI:** 10.1371/journal.pone.0252545

**Published:** 2021-06-23

**Authors:** Matthew Etterson, Nathan Schumaker, Kristina Garber, Steven Lennartz, Andrew Kanarek, Jennifer Connolly

**Affiliations:** 1 US Environmental Protection Agency, Office of Research and Development, Center for Computational Toxicology and Exposure, Great Lakes Toxicology and Ecology Division, Duluth, MN, United States of America; 2 US Environmental Protection Agency, Office of Research and Development, Center for Public Health and Environmental Assessment, Pacific Ecological Systems Division, Corvallis, OR, United States of America; 3 US Environmental Protection Agency, Office of Pesticide Programs, Environmental Fate and Effects Division, Washington, DC, United States of America; University of Brasilia, BRAZIL

## Abstract

Pesticides are used widely in agriculture and have the potential to affect non-target organisms, including birds. We developed an integrated modeling system to allow for spatially-explicit evaluation of potential impacts to bird populations following exposures to pesticides. Our novel methodology builds upon three existing models: the Terrestrial Investigation Model (TIM), the Markov Chain Nest Productivity Model (MCnest), and HexSim to simulate population dynamics. We parameterized the integrated modeling system using information required under the Federal Insecticide, Fungicide, and Rodenticide Act, together with species habitat and life history data available from the scientific literature as well as landcover data representing agricultural areas and species habitat. Our case study of the federally threatened California Gnatcatcher (*Polioptila californica*) illustrates how the integrated modeling system can estimate the population-scale consequences of pesticide applications. We simulated impacts from two insecticides applied to wheat: one causing mortality (survival stressor), and the other causing reproductive failure (reproductive stressor). We observed declines in simulated gnatcatcher abundance and changes in the species’ distribution following applications of each pesticide; however, the impacts of the two pesticides were different. Our methodology attempts to strike a balance between biological realism and model complexity and should be applicable to a wide array of species, systems, and stressors.

## Introduction

Pesticides are used widely in urban, suburban, and rural environments and have the potential to affect organisms other than targeted pests [1–4]. From 2008–2012, the most recent years for which statistics are available, over 1 billion lbs of pesticide active ingredient (a.i.) were sold annually in the United States [5]. Insecticides accounted for about 64 million lbs a.i. sold, of which approximately 64%, or 38 million lbs a.i./yr were used in the agricultural sector. At least 29 different modes of action (MOA) have been identified for agricultural insecticides, which target different aspects of the biology and physiology of target pests, including the nerve and muscular systems, growth, respiration, and gut function [6].

To identify and characterize risk of pesticides to birds and other non-target organisms, the United States Environmental Protection Agency (USEPA) Office of Pesticide Programs (OPP) uses a tiered risk assessment process [7]. Current USEPA/OPP tiered risk assessment for birds starts with the T-REX model [8], which provides a conservative estimate of exposure through diet. Tier I assessments are based on risk quotients (RQs), which are calculated by dividing a conservative estimate of exposure by a threshold toxicity value representing mortality or sublethal effects (*e*.*g*., the median lethal dose or LD50; the median lethal concentration or LC50; the No Observed Adverse Effect Concentration or NOAEC; the No Observed Adverse Effect Level or NOAEL). RQs are compared to levels of concern (LOCs) to determine whether a pesticide use poses risks of concern for mortality, growth or reproductive effects. If RQs are below the acute and chronic LOCs, it is concluded that the insecticide does not pose a risk of concern to the species or taxon. In cases where a RQ exceeds a LOC, there is potential risk of effects, and a higher tier assessment may be warranted. For higher tier avian risk assessments, one option available for OPP is the Terrestrial Investigation Model (TIM, v.3.0) [9], a probabilistic model that focuses on acute exposures to birds. For chronic risks, the Markov Chain Nest Productivity Model (MCnest) [10, 11], a mechanistic model of avian breeding, is available to assess potential declines in the annual reproductive success of exposed bird populations. The T-REX, TIM, and MCnest models are focused on field-level exposures to birds and resulting effects. The models assess risks to individuals or small groups of birds (e.g., flocks) that are assumed to be exposed to a pesticide on a use site (e.g., wheat field) or adjacent area receiving spray drift.

Under Section 7 of the U.S. Endangered Species Act (ESA) [12–14] EPA makes effects determinations to assess whether a pesticide’s use may affect a listed (i.e., threatened or endangered) species, and, if so, the US Fish and Wildlife Service (FWS) or the National Marine Fisheries Service (NMFS) may evaluate if a pesticide’s use jeopardizes the persistence of that species. Among the tools and methods under development as part of this process are population models, which have long been recognized as a means for understanding the risks and consequences of adverse effects of pesticides on birds and other animals [15, 16]. A report from the US National Research Council (NRC) strongly endorsed the use of population models for assessing the risks to threatened and endangered species from exposure to pesticides (NRC 2013). Several population modeling approaches have been applied to avian chemical risk assessment, including matrix projection models [17] and individual-based models [18].

The California Gnatcatcher (*Polioptila californica*, hereafter CAGN) is an insectivorous songbird that occupies scrub habitats in the Baja peninsula of Mexico and southern California, ranging as far north as Ventura County, CA, USA [19]. CAGN was officially listed as threatened under the ESA in 1993. Principal threats to CAGN include habitat loss and fragmentation, increased frequency of fire, and nest parasitism by brown-headed cowbirds (*Molothrus ater*) [20]. The extent to which CAGN may be affected by exposure to insecticides has not been studied, though the California Department of Pesticide Regulation lists over 60 crops on which pesticides are used within the range of the species [21]. CAGN typically breed from late March through mid-July and their seasonal reproductive output usually includes multiple nesting attempts by a given breeding pair [19].

In this article, we describe a method for conducting spatially explicit population level risk assessment for birds. This work builds on that of Etterson and colleagues [4] who combined TIM and MCnest into a single unified model (TIM/MCnest) that considers both acute and chronic effects resulting from insecticide exposure during the northern temperate breeding season (approximately March–August). Here we show how breeding season predictions of insecticide effects from TIM/MCnest can be used in the HexSim modeling environment [22] to inform a spatially explicit population model for avian response to insecticide exposure. HexSim is user-friendly development platform within which researchers can construct spatially explicit and individual-based simulation models. Importantly, HexSim allows users to develop models that conform to data limitations, rather than requiring a fixed set of input parameters. We parameterize the models for two insecticides and apply them to study the potential effects of insecticide exposure on the California Gnatcatcher. Our objectives are 1) to create an integrated workflow that allows TIM/MCnest predictions of pesticide effects on avian reproductive success to be implemented within the HexSim environment; and 2) to apply the model to a federally listed species under realistic spatially referenced insecticide use patterns. Our presentation of the California Gnatcatcher simulations is intended as an example of how spatially explicit population level risk assessment for pesticides might be performed for a federally listed bird. Because the simulations we present are not, in their current form, intended to influence policy or management, the pesticides examined in this analysis are unnamed; however, their parameters are based on data available for specific insecticides. Thus, our third objective is to invite review from the risk assessment and modeling communities, in hopes that the best possible tools can be made available for use in avian pesticide risk assessments.

## Methods

### Models and integration

Salient features of each of the three simulation models are summarized in Table 1 and described in more detail below. Here we provide enough information to understand the basic workings of each model and supply additional references for those who wish to dig deeper.

**Table 1:** Characteristics of three simulation models that were deployed to create the integrated avian model system for pesticides.

	TIM	MCnest	HexSimPLE
**Programming environment**	C++	Matlab	C++
**Formalism**	Individual-based	Markov chain	IBM
**Processes**	• Chemical exposure • Acute effects • Local foraging	• Embryo and nest survival • Renesting rates • Breeding season • Reproductive effects	• Spatially explicit habitat distribution • Vital rates linked to habitat quality • Projection matrices used to simulate population dynamics • Density dependent dispersal •
**Sources of stochasticity**	• Probability of surviving exposure • Foraging location (on/off treated field) • Dietary residues • Body size	• Nest initiation probability • Daily nest survival	• Yearly environmental variability • Dispersal outcomes • Pesticide impact outcomes
**Prediction**	• Survival Probability	• Reproductive output	• Population growth • Population distribution
**url**	https://www.epa.gov/pesticide-science-and-assessing-pesticide-risks/models-pesticide-risk-assessment#terrestrial	https://www.epa.gov/chemical-research/markov-chain-nest-productivity-model	https://www.epa.gov/risk/hexsim-modeling-simulator-tool-hexsim

TIM v.3.0 (hereafter referred to as TIM) [9] is an individual-based exposure and effects model that predicts avian mortality attributable to acute pesticide exposure resulting from a realistic time-dependent pesticide use scenario. Detailed information on TIM has been provided elsewhere [4, 9] and we include only a brief summary of important features here. TIM accounts for exposure through diet, drinking water, inhalation, and dermal contact following insecticide spray application to crops using a 1-hour timestep. Bird foraging on treated fields is stochastic, following a correlated movement on and off treated areas, and cumulative exposure considers avian frequency of usage of field and adjacent habitats that receive spray drift. Exposure in adjacent areas receiving spray drift decreases with distance from the edge of the field, where exposure is estimated using an approach adapted from the AgDRIFT model [23]. TIM runs for a single growing season, which may or may not overlap with avian breeding. Pesticide application methods that may be modeled in TIM include aerial, airblast, ground broadcast, ground banded, and ground in furrow. For all these application methods, exposure can be assessed on the treated field and edge habitat where spray drift is transported. Important assumptions of TIM include:

1. Toxicity to simulated birds is represented by surrogate test species (often the most sensitive of species tested);
2. Dose is a function of diet, inhalation, drinking water, and dermal uptake, as well as elimination;
3. Intake rates are allometrically scaled to bodyweight;
4. Birds move on and off treated fields with varying fidelity based on species characteristics;
5. Birds follow a bimodal feeding pattern where peak feeding occurs soon after sunrise and before sunset;
6. Pesticide concentrations on food items conform to a lognormal distribution;
7. Acute toxicity (LD50) is scaled using body weight and, where available, empirical pesticide-specific scaling factors [24].

The Markov chain nest productivity model (MCnest) is a mechanistic Monte Carlo simulation model that estimates declines in reproductive success for temperate zone birds during the breeding season [10, 13]. The model uses a Markov transition matrix [25] to follow female birds as they complete a breeding cycle [26] of nest establishment, survival, nest failure, and re-nesting up to the end of a typical breeding season. The number of successful nests per breeding female in a single breeding season is tracked, along with the cause for each failed nest. Insecticide induced failures are modeled by the inclusion of daily exposure values from TIM. These values are compared to phase-specific NOAELs for different kinds of adverse effects that might be induced by insecticide exposure such as eggshell thinning, reduced egg viability, reduced hatching success, and increased abandonment [10, 27]. Populations with identical parameter values are simulated with replication to provide estimates of variability around model predictions. MCnest output consists of estimates of the expected number of successful young per female in a population exposed to a given insecticide use scenario. Bennett and Etterson [28–30] provide detailed information on MCnest, including its use, technical background, and species library. Important assumptions underlying MCnest are:

1. Females necessarily attempt to renest if there is sufficient time remaining in the breeding season;
2. Demographic parameters, such as nest survival rates, clutch size, and waiting periods post failure and post fledging are fixed for all individuals;
3. Nest failures occur as a unit (i.e., not on a per-egg basis), whether natural or induced by insecticide exposure;
4. Exceedance of phase-specific toxicity endpoints results in complete nest failure;
5. NOAELs from the avian reproduction test are generally applicable across species.

Assumptions (3) and (4) have been criticized for causing MCnest to over-predict pesticide impacts on birds [31]. Although these assumptions do *usually* result in conservative predictions, they do not always do so. Their importance in light of the results presented herein is reviewed in the Discussion below.

TIM and MCnest were integrated within the Matlab [32] programming environment by having MCnest call TIM as a subroutine [4]. These integrated simulations first run TIM on adults to generate exposure and mortality results for each simulated breeding female; reproduction is then simulated using MCnest conditional on the mortality and exposure profiles generated from TIM. The integrated model is available online at: https://www.epa.gov/chemical-research/markov-chain-nest-productivity-model.

**Fig 1:**
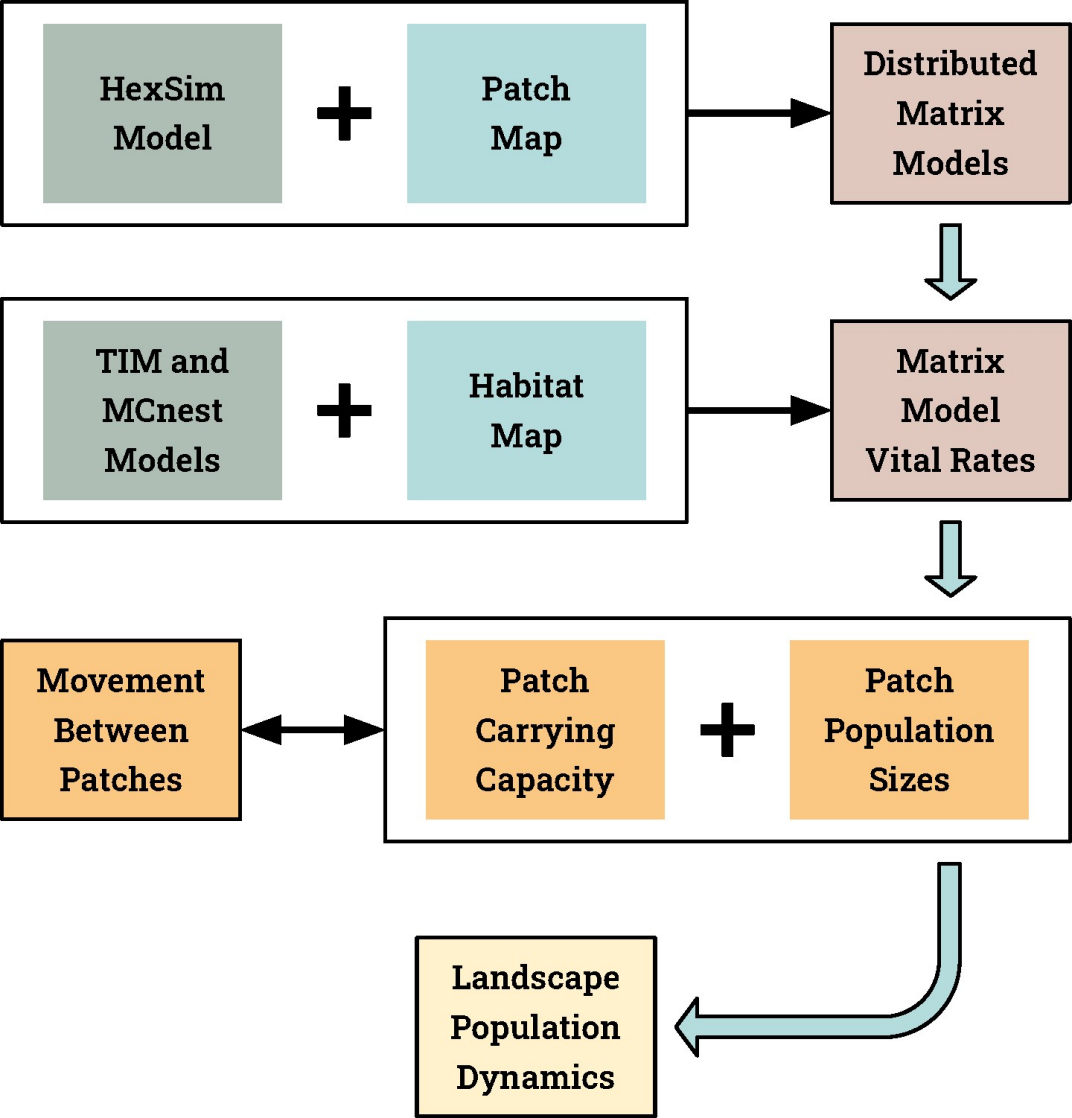
Conceptual model describing the integration of TIM/MCnest output (pesticide survival and fecundity values) with HexSim. Our HexSimPLE model instructs HexSim to assign individual projection matrix models to each patch in a patch map. Matrix survival and reproductive rates are then inferred from habitat quality and modified externally by TIM and MCnest based upon pesticide exposure. HexSimPLE then instructs HexSim to perform matrix multiplication, and to simulate density dependent movement between individual patches. Ultimately, population dynamics emerge from the spatially linked array of matrix models.

HexSim is a spatially explicit, individual-based model (IBM) designed for simulating terrestrial wildlife population dynamics and interactions. For this work, we used HexSimPLE (HexSim Populations Linked by Emigration), which is a customizable hybrid matrix/IBM constructed within HexSim and intended for use in developing parsimonious rapid response models (more detail provided below). HexSim and HexSimPLE are both available from www.hexsim.net. Our HexSimPLE gnatcatcher model consisted of a spatial array of 2-stage projection matrix population models [33] linked by spatially-explicit individual-based density-dependent dispersal (Fig 1). Model inputs included habitat and stress (insecticide) maps, a patch-map used to distribute individual population projection matrices across the landscape, stage-specific vital rates, and a few additional parameters used to specify carrying capacity and movement ability. Insecticide impacts on gnatcatcher vital rates were captured in the stress maps, which in-turn impacted individual matrix elements.

In the simulations described below, our patch-maps contained >24K 79 ha patches (and thus individual projection matrices), except along the range boundaries where patches were clipped and therefore somewhat smaller in size. We used two age-classes, juvenile (*j*) and adult (*a*), with the juvenile period lasting from the time of fledging up to the first breeding season at 1 year. Within patches, habitat quality (0 ≤ *q* ≤ 1) modified vital rates via a coefficient (*c*) that ranges from 0–1 according to the following function: *c* = 1−(1−*q*)^*α*^, where *α* is a user specified slope for the coefficient. For our simulations below, we used values of *α =* 3 for fecundity and *α =* 5 for survival, which were determined by tuning the model in the absence of simulated insecticides to produce range-wide population sizes at steady state that were roughly consistent with previous census data [34, 40]. Population size estimates obtained this way represent a hypothetical upper limit reflecting assumptions that detection rates were perfect and the pesticides were absent.

### Demography and environmental stochasticity

Demographic processes were modeled separately within each patch by using matrix multiplication to determine annual patch-specific population size, as a vector of age classes. Dispersal was limited to juveniles and modeled as a separate individual-based process (see Dispersal Model below). Therefore, aside from the spatially explicit dispersal step, patch-specific dynamics in ideal habitat (*q* = 1) can be described by the following equation:

$$N_{t+1}=[\begin{array}{cc} s_{j}f & s_{a}f\\ s_{j} & s_{a} \end{array}]N_{t},\;\mathrm{where}\;N_{t}=[\begin{array}{c} n_{j}(t)\\ n_{a}(t) \end{array}]$$ (1)

In Eq (1) *n*_*j*_(*t*) is the number of juvenile birds (age-class 1) at time *t* and *n*_*a*_(*t*) is the number of adult birds (age-class 2) at time *t* in the habitat patch. Fecundity (*f*), the number of female offspring fledged per female is the same for both age classes, *s*_*a*_ is the annual survival rate of adult birds and *s*_*j*_ is the annual survival rate of juvenile birds. When habitat is not perfectly suitable, the demographic parameters *s*_*a*_, *s*_*j*_, and *f* are multiplied by patch-specific vital rate coefficients (*c*) that reflect expected reductions (from their maximum) due to lower habitat quality. Eq (1) assumes a post-breeding census, which proved convenient for the integration of TIM/MCnest output, but HexSimPLE can also employ a pre-breeding census (or multiple censuses) as well. When a census is taken just before the breeding pulse, the youngest individuals will have been alive for almost a full year, and thus have already been subjected to mortality. This is referred to as a pre-breeding census. When the census is taken just after the breeding pulse, the youngest individuals will be at the beginning of their lives, and not yet have experienced any mortality. This is referred to as a post-breeding census.

To introduce insecticide effects in HexSimPLE we modified Eq (1) to incorporate mortality induced by the stressor and informed by the output of TIM/MCnest as an additional survival term, *s*_*p*_ = probability of mortality due to the patch-specific insecticide exposure:

$$N_{t+1}=\left[\begin{array}{cc} s_{p}s_{j}f & s_{p}s_{a}f\\ s_{p}s_{j} & s_{p}s_{a} \end{array}\right]N_{t}^{T}=\left[\begin{array}{cc} s_{j}f & s_{a}f\\ s_{j} & s_{a} \end{array}\right]s_{p}N_{t}^{T}$$ (2)

When there is no insecticide exposure, or no mortality due to insecticide exposure (*s*_*p*_ = 1), Eq (2) is equivalent to Eq (1). The insecticide survival term (*s*_*p*_) and fecundity term, *f*, are TIM/MCnest outputs reflecting expected survival and reproductive success during the breeding season under given insecticide exposure conditions [4, 11]. The survival terms (*s*_*j*_ and *s*_*a*_) are background rates of survival for juveniles and adults, respectively. By taking the product of the these and *s*_*p*_ we assume that reduced survival due to insecticide exposure is an independent competing risk. As in Eq (1), the vital rates in Eq (2) would be modified by habitat quality coefficients (*c*) to reflect further reductions due to habitat quality. Finally, for all models, an additional 10% variability was introduced to the three basic vital rates (*s*_*a*_, *s*_*j*_, and *f*) to account for environmental stochasticity. A value of 10% was chosen because it is large enough to represent actual stochasticity, but not so large as to mask the model’s response to our treatments. For simplicity of illustration, we assumed perfect correlation between the effects of stochasticity on survival and fecundity, but this assumption is not required by the model.

### Life-history data

To obtain life history data for CAGN to parameterize TIM, MCnest, and HexSimPLE we consulted Akçakaya and Atwood [34] and Atwood and Bontrager [19] and followed citations therein to the original sources. We also consulted primary literature and USFWS documents [20]. Table 2 gives the vital rates we used for CAGN in perfect habitat (*q* = 1).

**Table 2:** Demographic rates for CAGN in the best habitat (*q* = 1) in the absence of insecticide exposure.

Rate	Value
Annual juvenile survival (*s*_*j*_)	0.4314
Annual adult survival (*s*_*a*_)	0.5200
Annual fecundity (*f* = female offspring/female)	2.2600

### Habitat suitability model

To model habitat suitability, we took the estimated U.S. range of CAGN from USFWS [35] (since modified slightly) and used the habitat model of Akçakaya and Atwood [34], which requires estimates of four descriptive habitat variables, CSS = amount of coastal sage scrub, ELV = meters elevation above sea level, DGR = distance to grassland, and DTR = distance to nearest pixel (900 m^2^) containing at least 10% tree cover. We summarized these habitat descriptors using National Gap Analysis Program Land Cover Data [36] from which we aggregated 9 macro-group habitat classes (California Chaparral, California Coastal Scrub, Warm Interior Chaparral, Cool Interior Chaparral, Warm Pacific Coastal Beach Dune and Bluff Vegetation, Mojave-Sonoran Semi-Desert Scrub, North American Warm Desert Alkaline-Saline Semi-Desert Scrub, Great Basin Saltbrush Scrub, and Great Basin & Intermountain Tall Sagebrush Shrubland & Steppe) into a single coastal sage scrub (CSS) habitat class. We used the logistic regression coefficients of Akçakaya and Atwood [34] (their Table 3) in ArcMap 10.3.1 [37] to produce a map of habitat quality (0 ≤ *q* ≤ 1) per pixel across the entire U.S. range of CAGN. After exporting the vector habitat map as a raster image, we imported it into HexSim–a process that involved resampling the data at the scale of an individual hexagon (0.86 ha in this case). We then superimposed a regular grid of habitat patches across the U.S. range of CAGN, with each habitat patch containing up to 91 hexagons (~79 ha), unless clipped by the range boundary. Finally, per-patch habitat quality was set equal to the mean value taken over all patch hexagons. Per patch carrying capacity was set to K = 0.062 CAGN/hexagon ≈ 14 ha/ territory, which is slightly larger than average territory size [19]) but well within the range of reported values (2–18 ha) [38].

**Table 3:** Primary toxicity values and application information for insecticides modeled using TIM/MCnest.

Attribute	Reproductive Stressor	Survival Stressor
**LD50**^1^ **(**mg a.i./kg-bodyweight)	5000^m^	359^q^
**LC50**^1^ **(**mg a.i./kg-diet)	2354^q^	3497^q^
**NOEC**^1^ **(**mg a.i./kg-diet)	4.62^m^	358^q^
**Reproductive effect (reduction in)**	eggs laid	eggs laid, egg viability, eggshell thickness
**Maximum application rate (lbs a.i./acre)**	0.03	3
**Minimum application interval (d)**	5	7

^1^Tested species

^m^ = mallard

^q^ = northern bobwhite

### Dispersal model

CAGN dispersal was limited to juveniles, who moved according to the following rules. If patch-specific population size (N) > patch-specific carrying capacity (K), then N − K juveniles must disperse (otherwise they remain in their natal patch). For dispersers, maximum dispersal distance is drawn from a uniform distribution ranging from 0.5 to 10 km. Individuals disperse according to a highly autocorrelated random walk, stopping when they reach a patch with N < K. If no such patch is encountered by the time they reach maximum dispersal distance, then they stop anyway. This dispersal mechanism produced simulated dispersal path lengths that were lognormally-distributed. In unusual cases where path length constraints result in patches with N > K after dispersal, then juvenile CAGN are culled until N = K. By design, the number of adults will never exceed K in any patch, so culling of adults is not required. The overall CAGN simulation was calibrated by modifying the dispersal distribution, the autocorrelation parameter, carrying capacity, and habitat exponent (*α* in Eq 1) to get to a plausible spatial distribution with equilibrium number of CAGN pairs < 5,000, as per Partner’s-in-Flight US population estimate for CAGN [39].

### Insecticides and model applications

We developed model applications by simulating the use of two acetylcholinesterase-inhibiting insecticides on wheat crops within the range of CAGN. Simulations for the two insecticides were independent of each other. The insecticides were assumed not to affect carrying capacity, nor did we model indirect effects (e.g., prey reduction). We obtained spatial coverages of crop distributions from USEPA [12] and assumed that a given insecticide was used on 100% of the wheat crop, though potentially applied on different dates. Wheat was chosen as it is found in areas adjacent to the habitat of CAGN; however, other crops (e.g., lettuce, tomatoes) that potentially receive insecticide exposures also occur near the habitat of CAGN. Data on the effects of the two insecticides on birds were taken from information submitted in support of FIFRA pesticide registrations (Table 3). We excluded the names to emphasize that the focus of this work is on model development and procedure, not the actual effects of any specific products. In this case study, one pesticide was chosen because of individual-level risk concerns for mortality after acute exposures (hereafter survival stressor), and the other has risk concerns for reproductive effects following chronic exposures (hereafter reproductive stressor). Complete parameter sets used for TIM/MCnest are provided as S1 File. We used pesticide labeling information to determine the maximum application rates and assumed that growers could divide the maximum a.i. (lbs/acre) evenly into 2, 3, or 4 applications separated by the minimum interval between applications. For a given combination of insecticide and number of applications we created a hypothetical moving window of application dates by shifting the date of each application by 1 week and running TIM/MCnest to generate a curve of TIM/MCnest-predicted effects for application scenarios indexed by date of first application. We thus generated three such curves for each insecticide, corresponding to 2, 3, or 4 total applications.

These curves were then used to generate a spatially explicit distribution of insecticide effects by randomly assigning an application scenario (combination of date of first application and number of applications) to each habitat patch and using linear interpolation from the effects curves (produced by TIM/MCnest, and described above) to estimate fecundity or survival associated with that habitat patch and application scenario. In the above procedure, the proportion of each habitat patch planted in wheat was used to modify the expected effects of the insecticide on patch-specific vital rates using a binomial mixing model:

$$E(v)=pv_{e}+(1-p)v_{u}$$ (3)

In Eq (3) *v*_*e*_ = vital rate (fecundity or survival) predicted by TIM/MCnest under exposure conditions for the supplied patch-specific application date and rate, *p* = proportion of patch cultivated in wheat, and *v*_*u*_ = value of a vital rate that in the absence of exposure in that particular habitat patch (i.e., taking into account habitat quality, *q*). For CAGN, unexposed survival *s*_*u*_ = 1 and fecundity *f*_*u*_ = 2.26 female fledglings/female in the best habitat (*q* = 1). We generated 100 realizations of the random distribution of insecticide usage calculated in this way and then resampled those 100 realizations to produce 10 trials for each insecticide. For each trial we randomly selected 50 realizations of the insecticide use pattern and used these as a fixed 50-year time-series. Each trial consisted of ten replicate simulations of 50 years of CAGN population growth, using the fixed time-series of insecticide use maps. To create a new trial, we randomly selected another 50 realizations (with replacement) from the 100 generated use maps, and repeated the ten replicate simulations.

## Results

**Fig 2:**
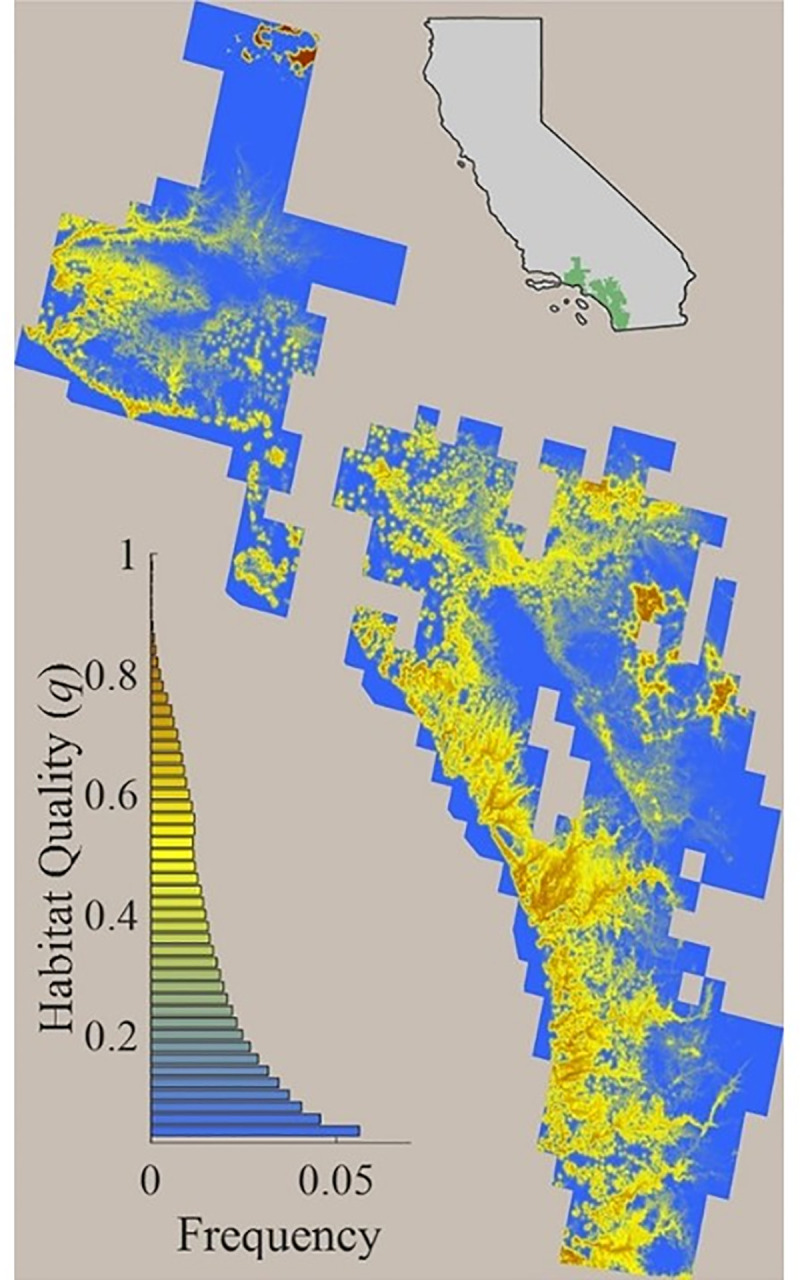
Predicted habitat quality for CAGN across the species’ range. Habitat quality values of 1 (red) indicate highest suitability for species.

Across the range of CAGN, modeled habitat quality varied considerably (Fig 2). Except for some small inland patches, the largest and highest quality habitat patches were predicted to occur in coastal areas. Approximately 13% of the land within the CAGN range [35] was classified as potentially suitable (*q* > 0.5) and mean habitat quality among suitable hexagons (79 ha ea.) was 0.63 (+/- 0.09 SD, n = 275,110). Habitat quality was negatively correlated with frequency of occurrence (Fig 2, lower inset), and high-quality habitat (*q* > 0.75) accounted for only about 1.5% of suitable habitat (*q* > 0.5).

When the vital rates of Table 2 are used in Eq (1), they give an estimate for density-independent finite rate of increase (λ) of 1.495, clearly demonstrating that, in ideal habitat, the species would be expected to exhibit strong positive population growth. With habitat exponents (α) of 3 and 5 for fecundity and survival, respectively, the transition between source and sink (λ = 1) would occur when habitat quality is approximately *q* ≈ 0.32.

**Fig 3:**
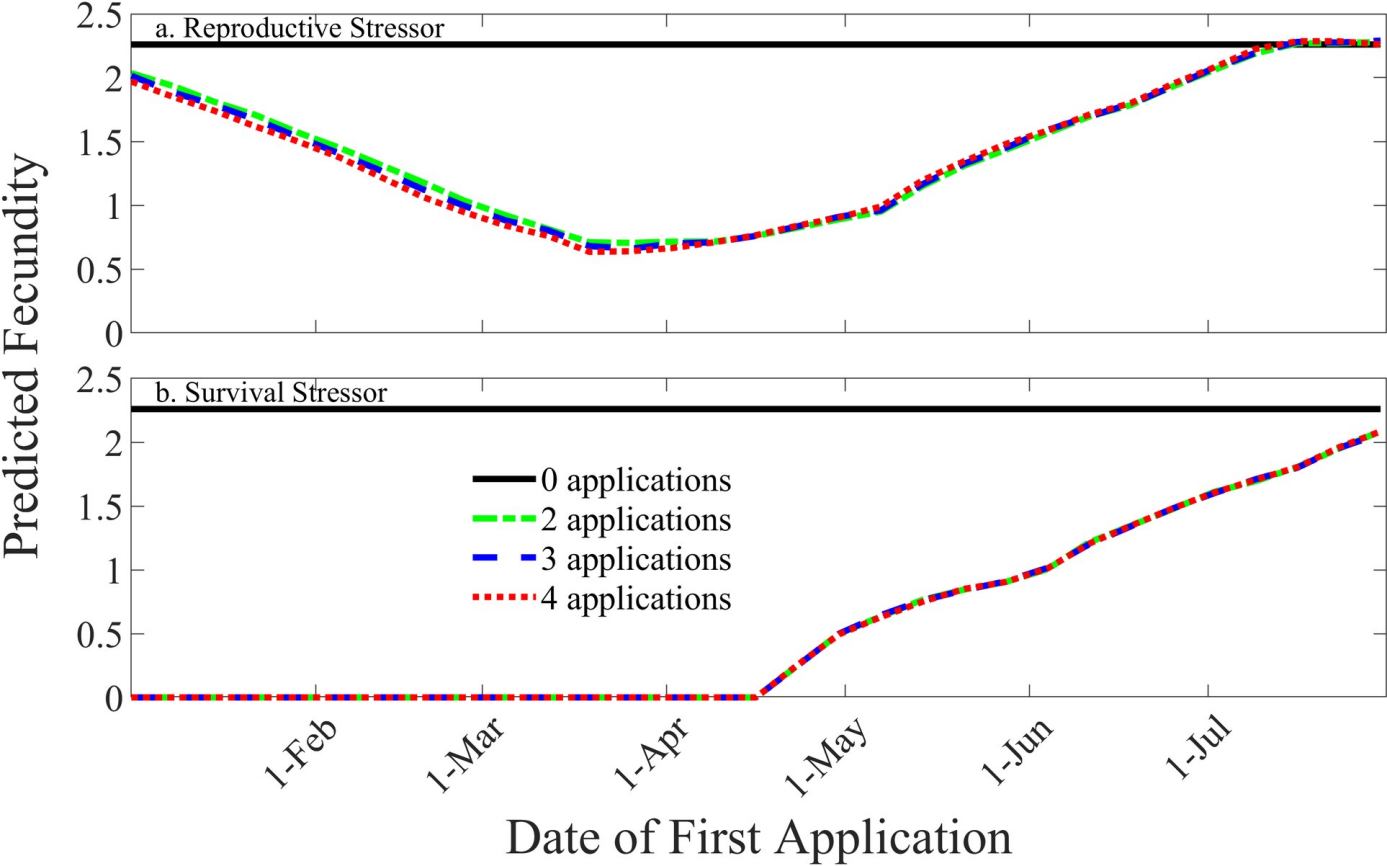
Effects of date of first application and number of applications on CAGN fecundity a. reproductive stressor, b. survival stressor. For both chemicals, the strong overlap indicates very little additional effects on fecundity due to repeated applications.

Predicted survival (*s*_*p*_) from TIM/MCnest contrasted sharply between the two modeled insecticides. All survival stressor scenarios resulted in predicted pesticide mortality probability (1-*s*_*p*_) of 1 for all exposed individual CAGN. Conversely, all reproductive stressor scenarios resulted in a predicted pesticide mortality probability of 0 for exposed individuals. In contrast, predicted seasonal productivity for exposed CAGN was eliminated by exposure to both insecticides (Fig 3) because all birds died under the survival stressor and all birds experienced zero fecundity under the reproductive stressor. Therefore, all TIM/MCnest predictions of effects of the reproductive stressor on fecundity are attributable to sublethal reproductive effects observed in the avian reproduction test results (Table 3) whereas all TIM/MCnest predictions of effects of the survival stressor on fecundity are attributable to lethal effects. Avian reproduction test results for this chemical do show reductions in eggshell thickness, number of eggs laid, and proportion of viable eggs set at the highest test concentration (1,260 mg/Kg, diet, Table 2, S1 File), but any effects on fecundity are made irrelevant by the prediction of 100% mortality, given exposure, in the modeled scenarios.

Date of first application had a strong effect on predicted fecundity, whereas the number of applications affected predicted fecundity little (Fig 3A, reproductive stressor) or not at all (Fig 3B, survival stressor). Seasonal patterns in model predictions for reductions in fecundity differed strongly between the two insecticides (Fig 3). For the reproductive stressor, TIM/MCnest predicted reductions in fecundity associated with applications as early as 1 January (Fig 3A), but not complete loss of reproductive output for the full season. This result is due to the presumed persistence of this chemical in the environment (lacking an estimate of the degradation half-life, the default value of 35 days was used). In contrast, TIM/MCnest predicted a complete loss of reproductive success from early applications of the survival stressor due to the lethal nature of exposure. For both insecticides, as the date of first application moved beyond the window of nest initiation for CAGN, which ends in roughly mid-July, predicted fecundity increased because modeled birds were able to successfully reproduce before experiencing any exposure to the insecticide.

**Fig 4:**
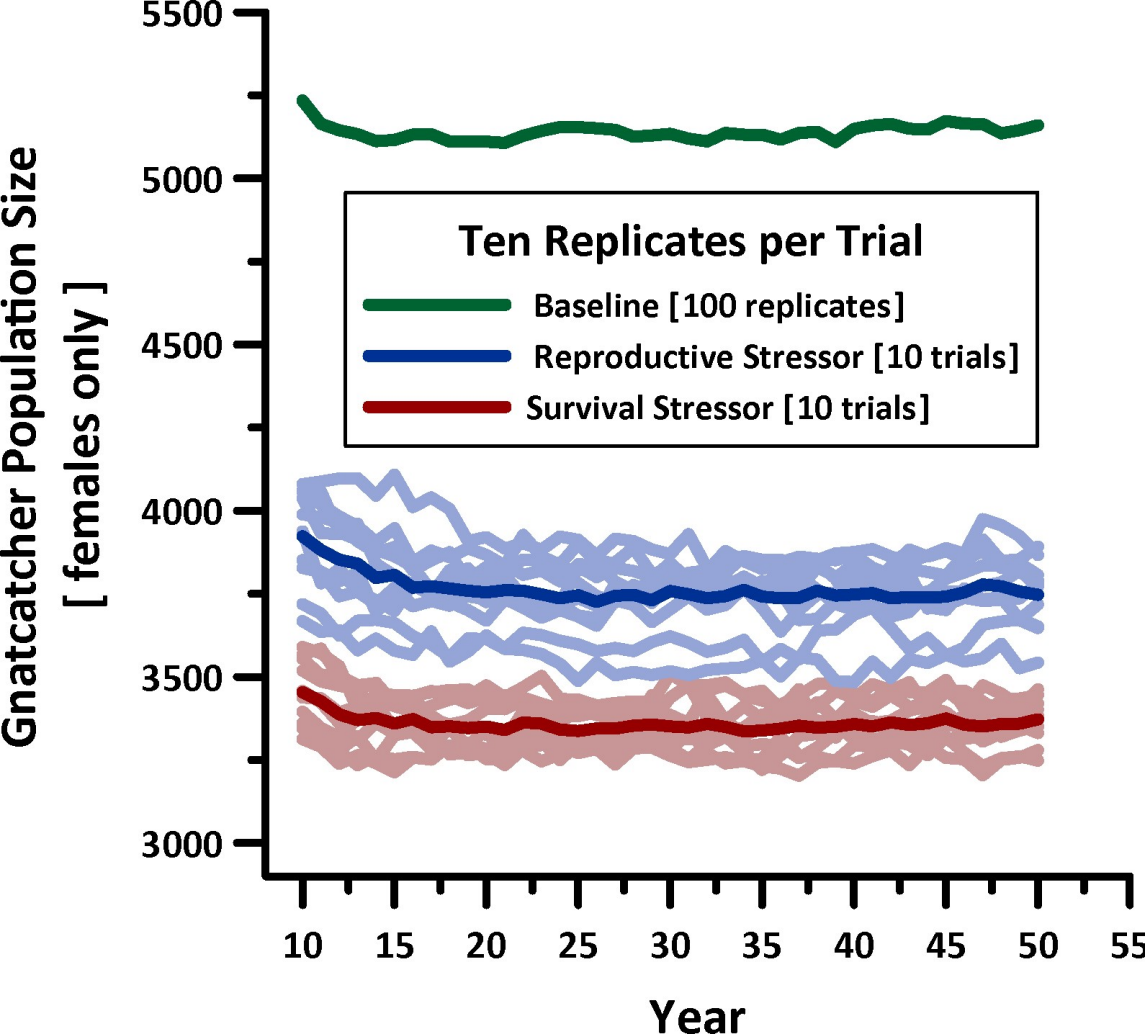
Time-series of CAGN abundance over 40 years with exposure. Variation around trend lines is due to application of +/- 10% environmental stochasticity.

HexSimPLE simulations of CAGN over 50 years showed differences between the two insecticides and control (Fig 4). Under control simulations (no insecticide) CAGN persisted at approximately 5,000 breeding pairs. Model runs predicted declines under both insecticides, with a new equilibrium being reached after approximately 15–20 years (Fig 4). Predicted population size at year 50 under the survival stressor was lower than under the reproductive stressor due to the high mortality associated with the former insecticide. Individuals exposed to the survival stressor were predicted to be permanently removed from the population, whereas individuals exposed to the reproductive stressor, though they may have lost some or all expected fecundity for a given year, could nevertheless survive and attempt to breed the following season (or later the same season, if time permits).

**Fig 5:**
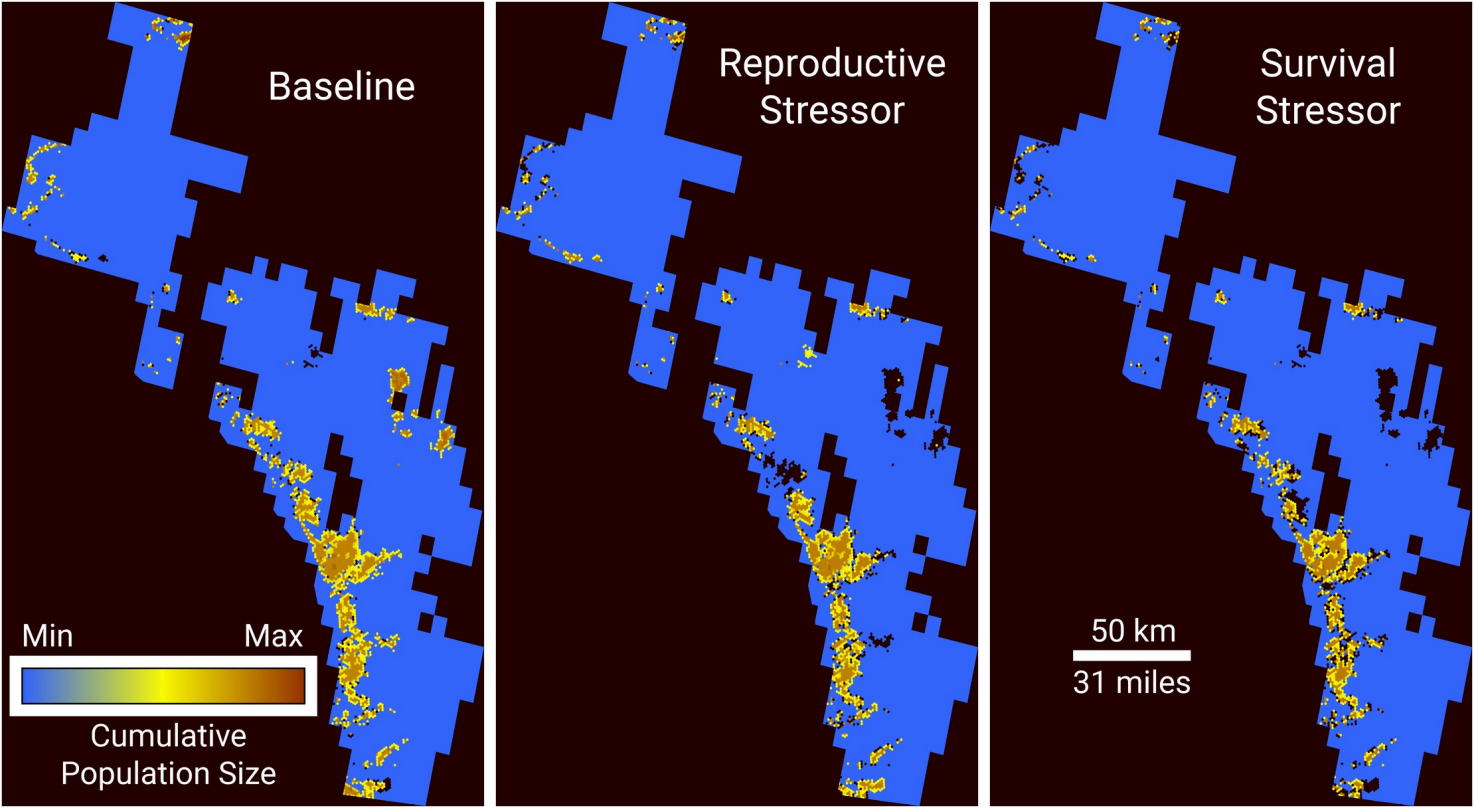
Predicted cumulative abundance of CAGN under baseline conditions and under the two simulated insecticide use scenarios.

Fig 5 shows the predicted change in distribution of CAGN with exposure to both insecticides compared to control. The spatial distribution of the bird remains relatively constant, with some losses of habitat patches, particularly in the inland sites. However, most patches show some predicted contraction under each insecticide and even large contiguous patches show some reduction in cumulative abundance.

## Discussion

Our results suggest that if both chemicals are used as described in this manuscript, then the survival stressor is predicted to have a greater impact on CAGN than the reproductive stressor. This result makes sense from a strictly mathematical point of view. The finite rate of increase (*λ* = dominant eigenvalue) for the matrix presented in Eq (1) is *λ = s*_*a*_ *+ s*_*j*_*f* (other symbols as previously defined). Parameterized with the data from Table 2 (i.e., for ideal habitat), the sensitivity of *λ* to changes in *s*_*a*_ is 1, whereas the sensitivity of *λ* to changes in *f* is 0.4314. Thus, the underlying demographic model suggests that the population growth rate should be more than twice as sensitive to changes in adult survival than to changes in fecundity, and this is reflected in the greater predicted population declines resulting from survival stressor exposure. However, this result should be interpreted with caution. The elasticities [33] of λ to changes in adult survival versus fecundity are reversed (0.35 vs. 0.65) suggesting that proportional changes in fecundity would have a greater effect on population growth than proportional changes in adult survival.

Our simulation results in the absence of insecticides differ strongly from those of Akçakaya & Atwood [34], who predicted decline and a high risk of extinction of CAGN. They further predicted that the studied Orange County population would fall below 60 individuals with 19% probability within 20 years and with 76% probability within 50 years. A more recent study by Winchell and Doherty [40] estimated the combined Orange County and San Diego County population on public lands to be 1,324 (976–1,673, 95% CI) [20, 40]. We calibrated our model to equilibrate at around 5,000 pairs across all habitat in the absence of insecticide exposure.

Our results differ from those of Akçakaya & Atwood [34] in part because we used vital rates that are at the high end of their ranges. Thus, for example our survival rate estimates (*s*_*j*_ = 0.4314 and *s*_*a*_ = 0.52, Table 2) are the highest of those rates reported by Akçakaya & Atwood [34]. Similarly, our fecundity rates derived from MCnest without insecticide applications were 2.26 female offspring/female, which closely matches the highest fecundity value (2.3) reported by Akçakaya & Atwood [34]. Thus, our vital rates are optimistic, but they apply only in habitat receiving a habitat quality score of q = 1, which represents a miniscule proportion of the total available habitat. The lowest rates reported by Akçakaya & Atwood [34] still occur in our model, but are associated with poor habitat quality scores (q < 0.5). Further, our vital rates (Table 2) are for parameterizing the density-independent matrix that describes within-patch dynamics. Once patch-specific carrying capacity is reached, juvenile survival declines as juveniles disperse from their natal patch, some of whom will fail to locate a suitable breeding patch, and are culled. Thus, our reported juvenile survival rate (*s*_*j*_) doesn’t include the contribution of density dependence to juvenile mortality, which is an emergent property of habitat quality, carrying capacity, and dispersal in our HexSimPLE model.

Another reason we chose the higher rates reported by Akçakaya & Atwood [34] is that in so doing we minimize the potential for double-counting the effects of insecticides (both of which were already registered and in use when the reported studies were conducted). In any case, our intent here is not to improve upon existing vital rate estimates, previous predictions of population trajectory, or population size for CAGN. Rather, we hope to build upon previous work and demonstrate how the TIM/MCnest/HexSim model, together with available information on vital rates, habitat quality, and population size for CAGN can be used to better understand and interpret the risk of pesticide exposure and effects to this federally threatened species.

Many sources of variability contribute to our simulation results (Table 1), including stochastic survival (TIM) and reproduction (MCnest), variable pesticide exposure and tolerance (TIM) among individuals, habitat heterogeneity (HexSim), probabilistic dispersal (HexSim), and environmental stochasticity (HexSim). With this demonstration we have not tried to quantify the contributions of these sources of uncertainty, though that remains an interesting avenue for further research.

Recently USEPA conducted a biological evaluation of several organophosphates under Section 7 of the ESA [12–14]. Thus, it is interesting to contrast those results with ours. Using TIM/MCnest, USEPA concluded that there were risks of mortality and/or reductions in reproductive success across all organophosphates examined in the study. Our results also show reductions in survival with the survival stressor modeled herein (and therefore reproductive success), but continued persistence of California Gnatcatchers. This helps to resolve an apparent paradox pointed out by Moore [31], that few populations could withstand such mortality, yet populations persist, even though these products have been used for many years. An important distinction is that the USEPA [12–14] risk characterizations consider exposed individuals only. Thus, when similar scenarios are run in a spatially explicit environment that includes habitat quality, a mix of exposed and unexposed individuals, and refuges from exposure, the populations are predicted to persist for at least 50 years in an apparently stable trajectory (Fig 4).

An interesting result of our simulations is that the number of applications had little or no effect on survival and/or fecundity of CAGN. This result is not general and should be interpreted with great caution. For the survival stressor, repeat applications had no effect because the model predicted 100% mortality upon the first application, due in large part to high predicted inhalation toxicity. For the reproductive stressor, repeat applications had little effect because of the 35d (default) half-life used for residues on dietary items. This had the effect of maintaining residue concentrations at toxic levels throughout the breeding season.

Perhaps the most useful aspect of the modelling framework we evaluate here will be the ability to evaluate the influence of factors surrounding how, when, and where an insecticide is applied on impacts to species of birds. For example, simulated application dates can be modeled in TIM/MCnest to determine when the risk of pesticide use is minimal. This may be most useful for insecticides that do not have high mortality risk but may cause sublethal reproductive effects. Similarly, the spatially explicit nature of HexSim can be used to explore the overlap of pesticide use, animal demography, species distribution, and critical habitat in a way that would protect essential resources, while perhaps allowing insecticide usage in marginal habitats. In addition, this framework allows for consideration of all relevant use sites in assessing exposures and risk to the population. In this example, wheat was simulated; however, exposures from other use sites that are adjacent to the resources used by CAGN could also be integrated into HexSimPLE, along with mortality and fecundity declines associated with those other uses. Although we did not do so in this case study, available crop specific usage data on the percent of treated area and actual application rates (e.g., California’s Pesticide Use Reporting data, https://www.cdpr.ca.gov/docs/pur/purmain.htm) could also be incorporated in order to evaluate the impact of actual application practices on risk.

While we believe our model has promise and offers substantial advances to current methods for avian pesticide risk assessment, we must also acknowledge that it has some important limitations. In particular, the assumptions listed in Methods should be carefully noted (we briefly touch on them here–they have been reviewed in more depth elsewhere [4, 28]). With some effort, some of those assumptions could be relaxed, though perhaps not all. One MCnest assumption was noted recently as an important limitation [31], which is that complete nest failure occurs with exceedance of phase-specific toxicity endpoints. As noted by Etterson et al. [4], relaxation of this assumption would be difficult, requiring quantitative description of the relationship between specific endpoints from toxicity tests and avian fledging success with exposure to insecticides. However, the toxicity tests, especially the avian reproduction test, simply weren’t designed to provide quantitative dose-response curves and would have to be significantly modified [28]. See references [4 and 10] for more in-depth discussion of the MCnest assumptions. The assumption of complete nest failure with endpoint exceedance is not universally conservative. A female who loses a complete nest attempt early to pesticide exposure, but subsequently renests successfully may experience greater reproductive success than a female who suffers low fledging success (*e*.*g*., raises one fledgling) from a successful nest attempt if doing so leaves insufficient time to renest.

Other important limitations to the model application described here include lack of connectivity between the Mexican and US CAGN populations. Our model includes no dispersal into or out of Mexico. We do not believe this to be a significant source of error in our simulations presented, or any plausible modifications thereof, primarily due to the relatively limited dispersal of CAGN [20, 34]. Our model also requires a habitat description. In the presented example, we had an existing high-quality habitat model available from the scientific literature upon which we could draw. For some listed species this will not be the case and a more qualitative model would have to be substituted. We do not know how such a model would perform in our linked modelling system, but we believe it is an important area for further research. Finally, an important limitation is that we have included only insecticide exposure as a single stressor (or arguably two stressors if poor-habitat quality is also considered), whereas many listed species are facing multiple threats, the combination of which result in the realized population trajectory of the taxon. For example, brown headed cowbird nest parasitism is an important stressor for CAGN that was not included in the example simulations provided herein, except as a component of background rates of nest failure. Similarly, insecticides may also affect birds indirectly by reducing the availability of invertebrate prey [41–43] (Boatman et al. 2004, Bright et al. 2008, Gibbons et al. 2014, Hallman et al. 2014), especially during the typical northern breeding season, a time of high energetic demand [44]. Our modeling system has the capacity for modeling nest parasitism and indirect effects as well as an arbitrary number of additional stressors, provided their spatial distribution can be described and their effects are known. In some cases, it may be argued (as we did immediately above concerning cowbird parasitism) that the effects of other stressors may already be incorporated into the background vital rates used for modeling. This will not be universally true and should be evaluated on a case by case basis. Another important and difficult example of multiple stressors that would require considerable further research to include would be the effects of exposure to multiple insecticides used simultaneously, or nearly so, at the landscape scale. Thus, while any number of complexities can be considered for inclusion, we believe these are best handled on a case-by-case basis, as most of these processes will require substantial effort to evaluate and parameterize.

## Conclusions

We have presented a spatially explicit population-level model for assessing risks of pesticides to passerine and near-passerine birds that use agricultural areas and adjacent habitats. We provided an example using the threatened California Gnatcatcher and showed how the model output could be used to better understand how the use of an insecticide across time and space may influence risk at the population level. However, this demonstration should be viewed as a beginning. A more thorough risk assessment using these models would include more stakeholders and a larger exploration of parameter space. Of course, a prerequisite to such an assessment involves identifying a modeling approach that is up to the challenge, but also scientifically sound and defensible. Here, we have described a new research tool that we believe has these very attributes. Our integrated models represent a major step towards the recent vision laid out by the National Academies [3] for a probabilistic approach to pesticide risk assessment for federally listed species.

## Supporting information

S1 File (DOCX)

## References

[1] Boatman ND, Brickle NW, Hart JD, Milsom TP, Morris AJ, Murray AWA, et al. Evidence for the indirect effects of pesticides on farmland birds. Ibis 2004; 146: 131–143. 10.1111/j.1474-919X.2004.00347.x

[2] Mineau P, Whiteside M. Lethal risk to birds from insecticide use in the United States–a spatial and temporal analysis. Environmental Toxicology and Chemistry 2006; 25: 1214–1222. doi: 10.1897/05-035r.1 16704051

[3] National Research Council. Assessing risks to endangered and threatened species from pesticides. Washington (DC): National Academies Press; 2013 195 p.

[4] Etterson MA, Garber K, Odenkirchen E. Mechanistic modeling of insecticide risks to breeding birds in North American agroecosystems. PLOS ONE 2017; 12: e0176998. doi: 10.1371/journal.pone.0176998 28467479 PMC5415183

[5] Atwood D, Paisley-Jones C. Pesticides industry sales and usage. USEPA/OPP/Biological and Economic Evaluation Division; 2017 [cited 21 Nov. 2020]. Available from: [https://www.epa.gov/sites/production/files/2017-01/documents/pesticides-industry-sales-usage-2016_0.pdf.

[6] Insecticide Resistance Action Committee [Internet] [cited 12 May 2017] Available from: http://www.irac-online.org/.

[7] USEPA. Overview of the Ecological Risk Assessment Process in the Office of Pesticide Programs. United States Environmental Protection Agency (USEPA). Environmental Fate and Effects Division. Office of Pesticide Programs; 2004 [cited 21 Nov. 2020]. Available from: http://www.epa.gov/espp/consultation/ecorisk-overview.pdf.

[8] USEPA. User’s Guide: T-REX Version 1.5 (Terrestrial Residue Exposure Model). US Environmental Protection Agency, Office of Pesticide Programs, Environmental Fate and Effects Division; 2012. Available from: http://www.epa.gov/oppefed1/models/terrestrial/trex/t_rex_user_guide.htm.

[9] USEPA. Technical description and user’s guidance document for the terrestrial investigation model (TIM) Version 3.0 Beta. US Environmental Protection Agency, Office of Pesticide Programs, Environmental Fate and Effects Division; 2015. Available from: http://www2.epa.gov/pesticide-science-and-assessing-pesticide-risks/models-pesticide-risk-assessment#tim.

[10] Bennett RS, Etterson MA. Incorporating results of avian toxicity tests into a model of annual reproductive success. Integrated Environmental Assessment and Management 2007; 4: 498–507. 10.1897/ieam_2007-029.1 18046799

[11] Etterson MA, Bennett RS. Quantifying the effects of pesticide exposure on annual reproductive success of birds. Integrated Environmental Assessment and Management 2013; 9: 590–599. doi: 10.1002/ieam.1450 23728843

[12] USEPA. Biological evaluation chapters for chlorpyrifos ESA assessment. US Environmental Protection Agency; 2016 [cited 2017 June 27]. https://www.epa.gov/endangered-species/biological-evaluation-chapters-chlorpyrifos-esa-assessment.

[13] USEPA. Biological evaluation chapters for diazinon ESA assessment. US Environmental Protection Agency; 2016 [cited 2017 June 27]. https://www.epa.gov/endangered-species/biological-evaluation-chapters-diazinon-esa-assessment.

[14] USEPA. Biological evaluation chapters for malathion ESA assessment. US Environmental Protection Agency; 2016 [cited 2017 June 27] https://www.epa.gov/endangered-species/biological-evaluation-chapters-malathion-esa-assessment.

[15] ECOFRAM. 1999. ECOFRAM Terrestrial Draft Report. Ecological Committee on FIFRA Risk Assessment Methods. US Environmental Protection Agency; 1999. [cited 11 May 2017] Available from: https://www.epa.gov/pesticide-science-and-assessing-pesticide-risks/ecofram-terrestrial-draft-report.

[16] Pastorok RA, Bartell SM, Ferson S, Ginzbug LR editors, Ecological Modeling in Risk Assessment: Chemical Effects on Populations, Ecosystems, and Landscapes. Boca Raton: CRC Press; 2001.

[17] Gleason TR, Nacci DE. Risks of Endocrine-Disrupting Compounds to Wildlife: Extrapolating from Effects on Individuals to Population Response. Human and Ecological Risk Assessment 2001; 7: 1027–1042.

[18] Topping CJ, Odderskær P. Modeling the influence of temporal and spatial factors on the assessment of impacts of pesticides on skylarks. Environmental Toxicology and Chemistry 2004; 23: 509–520. doi: 10.1897/02-524a 14982400

[19] Atwood JL, Bontrager DR. California Gnatcatcher (Polioptila californica). In: Rodewald PG, editor The Birds of North America. Ithaca: Cornell Lab of Ornithology; 2001; Retrieved from the Birds of North America: https://birdsna-org.bnaproxy.birds.cornell.edu/Species-Account/bna/species/calgna. doi: 10.2173/bna.574

[20] USFWS. US Fish and Wildlife Service. Coastal California gnatcatcher (Polioptila californica californica) 5 year review. US Fish and Wildlife Service, Carlsbad Fish and Wildlife Office, Carlsbad (CA). 2010; [Accessed 27 June 2017]. Available from: https://ecos.fws.gov/docs/five_year_review/doc3571.pdf

[21] Marovich RA, Kishaba S. Species by Commodity and Commodities by Species: An Index to Pesticide Use Sites (Commodities) That Occur in Proximity to Federally Listed, Proposed and Candidate Species in California. Sacramento (CA). Department of Pesticide Regulation California Environmental Protection Agency; 1997. cited 27 June 2017. Available from: http://www.cdpr.ca.gov/docs/endspec/espdfs/comxsp.pdf

[22] Schumaker NH, Brookes A. HexSim: a modeling environment for ecology and conservation. Landscape Ecology 2018; 33: 197–211. doi: 10.1007/s10980-017-0605-9 29545713 PMC5846496

[23] Teske ME, Bird SL, Esterly DM, Ray SL, and Perry SG. A User’s Guide for AgDRIFT 2.01: A Tiered Approach for the Assessment of Spray Drift of Pesticides, Regulatory Version. 2001; Continuum Dynamics Report No 01–02.

[24] Mineau P, Collins BT, Baril A. On the use of scaling factors to improve interspecies extrapolation of acute toxicity in birds. Regulatory Toxicology and Pharmacology 1996; 24: 24–29. doi: 10.1006/rtph.1996.0061 8921543

[25] Etterson MA, Bennett RS, Kirshner E, Walk J. Markov chain estimation of avian seasonal fecundity. Ecological Applications 2009; 19: 622−630. doi: 10.1890/08-0499.1 19425426

[26] Etterson MA, Ellis-Felege SN, Evers D, Gauthier G, Grzybowski JA, B.J. Mattsson, et al. Modeling fecundity in birds: conceptual overview, current models, and considerations for future developments. Ecological Modelling 2011; 222: 2178–2190.

[27] Bennett RS, Etterson MA. Selecting surrogate endpoints for estimating pesticide effects on avian reproductive success. Integrated Environmental Assessment and Management 2013; 9: 600−609. doi: 10.1002/ieam.1478 23913487

[28] Bennett RS, Etterson MA. Estimating pesticide effects on fecundity rates of wild birds using current laboratory reproduction tests. Human and Ecological Risk Assessment 2006; 12: 762–781.

[29] Bennett RS, Etterson MA. User’s Manual for Basic Version of MCnest–Markov Chain Nest Productivity Model. US Environmental Protection Agency; 2013. EPA/600/R/13/034. Available from: http://www.epa.gov/chemical-research/markov-chain-nest-productivity-model-documentation.

[30] Bennett RS, Etterson MA. Technical Manual for Basic Version of the Markov Chain Nest Productivity Model (MCnest). US Environmental Protection Agency; 2013. EPA/600/R/13/033. Available from: http://www.epa.gov/chemical-research/markov-chain-nest-productivity-model-documentation.

[31] Moore DR. The dangers of overestimating avian risk of pesticides. Integrated Environmental Assessment and Management 2017; 13: 542–551. 10.1002/ieam.1890 28440942

[32] Mathworks. 2016. Matlab R2016b. The Mathworks, Inc. Natick (MA); 2016.

[33] Caswell H. Matrix Population Models: Construction, Analysis, and Interpretation. 2nd Ed. Sunderland, MA: Sinauer; 2001.

[34] Akçakaya HR, Atwood JL. A habitat-based metapopulation model of the California Gnatcatcher. Conservation Biology 1997; 11: 422–434.

[35] USFWS. ECOS Environmental Conservation Online System US Fish and Wilde Service. https://ecos.fws.gov/ecp/species/8178.

[36] USGS. Gap Analysis Program (GAP). US Geological Survey, National Land Cover. [cited 2 May 2011]. https://www.usgs.gov/core-science-systems/science-analytics-and-synthesis/gap.

[37] ESRI. ArcGIS Desktop: Release 10.3.1 Redlands (CA). Environmental Systems Research Institute; 2015.

[38] Preston KL, Mock PJ, Grishaver MA, Bailey EA, King DF. California Gnatcatcher territorial behavior. Western Birds 1998; 29: 242–257.

[39] Rosenberg KV, Kennedy JA, Dettmers R, Ford RP, Reynolds D, Alexander JD, et al. Partners in Flight Landbird Conservation Plan: 2016 Revision for Canada and Continental United States. Partners in Flight Science Committee; 2016. Available: https://www.partnersinflight.org/what-we-do/science/plans/.

[40] Winchell CS, Doherty PF. Using California gnatcatcher to test underlying models of habitat conservation plans. Journal of Wildlife Management 2008; 72: 1322–1327.

[41] Bright JA, Morris AJ, Winspear R. A Review of Indirect Effects of Pesticides on Birds and Mitigating Land-management Practices. Royal Society for the Protection of Birds; 2008. RSPB Research Report No: 28.

[42] Gibbons D, Morrissey CA, Mineau P. A review of the direct and indirect effects of neonicotinoids and fipronil on vertebrate wildlife. Environmental Science and Pollution Research 2014; 22: 103–118. doi: 10.1007/s11356-014-3180-5 24938819 PMC4284370

[43] Hallman CA, Foppen RPB, van Turnhout CAM, de Kroon H, Jongejans E. Declines in insectivorous birds are associated with high neonicotinoid concentrations. Nature 2014; 511: 341–344. doi: 10.1038/nature13531 25030173

[44] Nagy LR, Holmes RT. Food limits the annual fecundity of a migratory songbird: an experimental study. Ecology 2005; 86: 675–681.

