# Supplementary material for: A spatially explicit model for estimating risks of pesticide exposure to bird populations
Source: PLoS One. 2021 Jun 23;16(6):e0252545. doi: 10.1371/journal.pone.0252545 (PMC8221516; doi:10.1371/journal.pone.0252545)
Supplement: S1 File — (DOCX) [file pone.0252545.s001.docx]

Supplementary Data for Etterson et al. “A spatially explicit model for estimating risks of pesticide exposure on bird populations”.

S1 Table. Life history data and sources for California Gnatcatcher for use in TIM/MCnest/HexSim

| Parameter |  | Value | Source |
| --- | --- | --- | --- |
| Initiation probability | p | 0.25 | MCnest default |
| daily nest mortality rate during laying & incubation | m1 | 0.043 | Grishaver et al. 1998 |
| daily nest mortality rate during nestling rearing | m2 | 0.019 | Grishaver et al. 1998 |
| date of first egg of first nest (dd-mmm) | T1 | 22 March | Grishaver et al. 1998 |
| date of first egg of last nest (dd-mmm) | Tlast | 13 July | Grishaver et al. 1998 |
| Egg mass (used to calculate rfg) |  | 0.99 | Hanna 1934 |
| length of rapid follicle growth period for each egg (d) | rfg | 3 | 2.8, using allometric eqn. of Alisauskas and Ankney 1992. |
| mean clutch size | clutch | 4 | Atwood & Bontrager 2001: 3.8 (Atwood 1988) USFWS 2010: Clutch size = 4 |
| mean intra-egg laying interval (days) | eli | 1 | Atwood & Bontrager 2001 |
| egg on which female typically begins incubation–penultimate (1) or last (0) | penult | penult | Atwood & Bontrager 2001 |
| duration from start of incubation to hatch (days) | I | 14 | Grishaver et al. 1998. |
| duration from hatch to fledging of nestlings (days) | N | 13 | Grishaver et al. 1998 |
| duration from nest failure until female new nest (days) | We | 1 |  |
| duration from fledging until new nest (days) | Wf | 15 | Atwood & Bontrager 2001 |
| Female Body Weight (g) | BdyWt | 5.9 | Atwood & Bontrager 2001 |
| Dietary proportions [short grass, tall grass, fruit, seeds, invertebrates] | Diet | [0,0,0,0,1] | Atwood & Bontrager 2001? |
| mean number of fledglings/ successful nest | fpsn | 2.24 | Grishaver et al. 1998:Table 2 |
| Annual adult survival rate | sa | 0.52 | Akcakaya and Atwood 1997 |
| Annual juvenile survival rate | sj | 0.4314 | Akcakaya and Atwood 1997 |
| Exponent for survival/habitat coefficient | α_s_ | 5 | Calibration parameter |
| Exponent for fecundity/habitat coefficient | α_f_ | 3 | Calibration parameter |
| Per hexagon carrying capacity | K | 0.68 | Calibration parameter |

S2 Table. TIM parameters used for CAGN simulations that were identical for both pesticides

| Parameter | Value |
| --- | --- |
| Model dietary exposure | yes |
| Model exposure through drinking from puddles | yes |
| Model exposure through drinking from dew | yes |
| Model exposure through dermal contact with foliage | yes |
| Model exposure through dermal contact with spray | yes |
| Model exposure off-field through spray drift | yes |
| Model exposure through vapor inhalation | yes |
| Model exposure through spray inhalation | yes |
| Number of applications | 3 |
| Application method (spray) | Aerial |
| Time of first application | 8:00 AM |
| droplet spectrum | very fine to fine |
| Spray height | 3 m |
| Spray duration (min) | 1.5 |
| Crop height (m) | 0.152 |
| Plant(crop) mass (kg/ha) | 39 |
| crop type | field |
| Fraction of edge habitat receiving spray drift | 1 |
| Length of in field buffer (feet) | 0 |
| fraction of organic carbon in soil | 0.015 |
| soil bulk density (kg/L) | 1.5 |
| Morning feeding start times: min and max | 4:00 am, 5:00 am |
| Morning feeding end times: min and max | 6:00 am, 10:00 am |
| afternoon feeding start times: min and max | 4:00 pm, 7:00 pm |
| afternoon feeding end times: min and max | 8:00 pm, 9:00 pm |
| Proportion of daily feeding taking place in morning: min and max | 0.4, 0.6 |
| Gorging factor | normal feeding |
| Contaminated fraction of food | 1 |
| Dislodgable foliar residue adjustment factor | 0.62 |
| Dermal adsorption fraction | 1 |
| avian acute inhalation LD50 (mg a.i.kg-bw) | unavailable |
| Chemical specific avian dermal LD50 | unavailable |
| Food matrix adjustment factor | 1 |
| ratio of juvenile to adult toxicity | 1 |
| Passerine vs. Non-passerine | 1 |
| Altricial vs. precocial | 1 |
| Body weight (g): mean, SD, min, max | 5.9, 0.6, 5.3, 6.4 |
| feeding category: ( insectivore, herbivore, granivore, omnivore) | insectivore |
| Fraction of each food item in diet [insects, seeds, fruit, grass, broadleaf] | [1,0,0,0,0] |
| Juvenile dietary proportions [insects, seeds, fruit, grass, broadleaf] | [1,0,0,0,0] |
| Resident status (field vs. edge) | edge |
| Respiratory physiology adjustment factor | 2.6 |
| Frequency on field [mean, min, max] | [0.1, 0, 0.2] |
| Fidelity factor | 0.6 |

S3 Table. TIM parameters that varied among pesticides

| Pesticide | Reproductive  Stressor | Survival  Stressor |
| --- | --- | --- |
| Rate of application #1 (lb a.i.A) | 0.0075 – 0.015 | 0.75 – 1.5 |
| Interval between app1 and 2 (days) | 5 | 7 |
| Rate of application #2 (lb a.i.A) | 0.0075 – 0.015 | 0.75 – 1.5 |
| Interval between app2 and 3 (days) | 5 | 7 |
| Rate of application #3 (lb a.i.A) | 0.0075 – 0.015 | 0.75 – 1.5 |
| Interval between app3 and 4 (days) | 5 | 7 |
| Rate of application #4 (lb a.i.A) | 0.0075 – 0.015 | 0.75 – 1.5 |
| Interval between app 4 and 5 (days) | 0 | 0 |
| Rate of application #5 (lb a.i.A) | 0 | 0 |
| Food item half-lives (days) | 35 | 6.1 |
| Pesticide half-life (days) in puddle | 76.2 | 15.9 |
| K_oc_ (Lkg-oc) | 333200 | 217 |
| K_ow_ | 1.00E+07 | 799 |
| Henry's law constant (atm*m^3^/mol) | 1.90E-07 | 1.20E-07 |
| solubility in water (mg a.i./L) | 0.005 | 145 |
| avian acute oral LD50 (mg a.i.kg/bw) | 50000 | 359 |
| Body weight of tested animals | 1049 | 195 |
| slope of avian oral LD50 | 10 | 9.42 |
| Mineau scaling factor | 1.15 | 1.15 |
| Rat inhalation LD50 (mg a.i.kg/bw) | 229 | 310 |
| rat acute oral LD50 (mg a.i.kg/bw) | 56 | 5400 |
| Hourly fraction of pesticide retained | 0.998 | 0.986 |

S4 Table. Avian toxicity and associated data

| Pesticide | Reproductive  Stressor | Survival  Stressor |
| --- | --- | --- |
| Reproduction Test species: | Mallard | Bobwhite |
| Dose levels: | 2 | 3 |
| Measured dietary conc. (mg/kg diet): | [4.62, 50.8] | [112, 358, 1260] |
| Average food consumption (g/bird/day): | [225.8, 218.3] | [24, 25, 23] |
| Average initial female body weight (g): | [962, 962] | [195, 194, 190] |
| Average initial male body weight (g): | [1087, 1075] | [193, 192, 193] |
| Average final female body weight (g): | [1135, 1113] | [250, 228, 202] |
| Average final male body weight (g): | [1249, 1251] | [221, 219, 206] |
| NOAELs |  |  |
| ^1^Number of eggs laid: | 1 | 2 |
| ^1^%Viable eggs of eggs set: | 2 | 2 |
| ^1^%Live 3-wk embryos of ^1^viable eggs: | 2 | 3 |
| ^1^%hatchlings of live 3-wk embryos: | 2 | 3 |
| ^1^%14-d chicks of ^1^hatchlings: | 2 | 3 |
| ^1^shell thickness: | 2 | 2 |
| ^1^prelaying female weight: | 2 | 3 |
| ^1^prelaying male weight: | 2 | 3 |
| Mineau scaling factor: | 1.15 | 1.15 |
| LD50 (mg/kg bwt): | 5000 | 359 |
| LD50 Body Weight (g) | 1049 | 195 |
| LD50 Slope | 10 | 9.42 |
| LD50 Test species: | Mallard | Bobwhite |
| LC50: | 2354 | 3497 |
| Fraction of LC50 (i.e., 0.1 or 0.5): | 0.5 | 0.5 |
| Mean body weight (g): | 17.4 | 22.5 |
| Mean food ingestion rate (g/d): | 14.96 | 7.7 |
| LC50 Test Species: | Bobwhite | Bobwhite |

^1^Numbers in these cells refer to testing levels and NOAECs for each endpoint. For example, the NOAEC for number of eggs laid for the survival stressor occurred at 358 mg ai/kg diet. Dietary doses (mg/kg body weight) are calculated from these food-concentration-based estimates, consumption rates, and body weights.

**Literature cited**

Akçakaya HR, Atwood JL. A habitat-based metapopulation model of the California Gnatcatcher. Conservation Biology 1997; 11: 422−434.

Alisauskas, RT. and Ankney CD. 1992. The cost of egg laying and its relationship to nutrient reserves in waterfowl. Pages 30-61 in: Batt BDJ, Afton AD, Anderson MG, Ankney CD, Johnson DH, Kadlec JA, Krapu GL, (eds.), Ecology and Management of Breeding Waterfowl. University of Minnesota Press, Minneapolis, MN.

Atwood JL. 1988. Speciation and geographic variation in Black-tailed Gnatcatchers. Ornithological Monographs 42.

Atwood, JL, Bontrager DR. 2001. California Gnatcatcher (*Polioptila californica*), The Birds of North America (P. G. Rodewald, Ed.). Ithaca: Cornell Lab of Ornithology; Retrieved from the Birds of North America: <https://birdsna-org.bnaproxy.birds.cornell.edu/Species-Account/bna/species/calgna>. DOI: 10.2173/bna.574

Grishaver MA, Mock PJ, Preston KL. 1998. Breeding behavior of the California Gnatcatcher in southwestern San Diego County, California. Western Birds 29:299-322.

Hanna, WC. 1934. The Black-tailed Gnatcatcher and the Dwarf Cowbird. Condor 36:89.

USFWS. US Fish and Wildlife Service. Coastal California gnatcatcher (Polioptila californica californica) 5 year review. US Fish and Wildlife Service, Carlsbad Fish and Wildlife Office, Carlsbad (CA). 2010; [Accessed 27 June 2017]. Available from: <https://ecos.fws.gov/docs/five_year_review/doc3571.pdf>.
